# Supplementary material for: OpenMM 7: Rapid development of high performance algorithms for molecular dynamics
Source: PLoS Comput Biol. 2017 Jul 26;13(7):e1005659. doi: 10.1371/journal.pcbi.1005659 (PMC5549999; doi:10.1371/journal.pcbi.1005659)
Supplement: S1 Source Code — (ZIP) [file pcbi.1005659.s002.zip › OpenMM-7.0.1-Source/docs/OpenMMDeveloperGuide.pdf]

OpenMM Developer Guide

Release 7.0

Peter Eastman

May 25, 2016

CONTENTS

1 Introduction **2**

2 The Core Library **3**

3 Writing Plugins **7**
- 3.1 Creating New Platforms . . . 7
- 3.2 Creating New Forces . . . 8

4 The Reference Platform **9**

5 The CPU Plaform **10**

6 The OpenCL Platform **11**
- 6.1 Overview . . . 11
- 6.2 Numerical Precision . . . 11
- 6.3 Computing Forces . . . 12
- 6.4 Nonbonded Forces . . . 13
- 6.5 Bonded Forces . . . 14
- 6.6 Reordering of Particles . . . 15
- 6.7 Integration Utilities . . . 15

7 The CUDA Platform **17**
- 7.1 Compiling Kernels . . . 17
- 7.2 Accumulating Forces . . . 17

Portions copyright (c) 2011-2016 Stanford University and the Authors

Contributors: Peter Eastman

Permission is hereby granted, free of charge, to any person obtaining a copy of this document (the "Document"), to deal in the Document without restriction, including without limitation the rights to use, copy, modify, merge, publish, distribute, sublicense, and/or sell copies of the Document, and to permit persons to whom the Document is furnished to do so, subject to the following conditions:

This copyright and permission notice shall be included in all copies or substantial portions of the Document.

THE DOCUMENT IS PROVIDED "AS IS", WITHOUT WARRANTY OF ANY KIND, EXPRESS OR IMPLIED, INCLUDING BUT NOT LIMITED TO THE WARRANTIES OF MERCHANTABILITY, FITNESS FOR A PARTICULAR PURPOSE AND NONINFRINGEMENT. IN NO EVENT SHALL THE AUTHORS, CONTRIBUTORS OR COPYRIGHT HOLDERS BE LIABLE FOR ANY CLAIM, DAMAGES OR OTHER LIABILITY, WHETHER IN AN ACTION OF CONTRACT, TORT OR OTHERWISE, ARISING FROM, OUT OF OR IN CONNECTION WITH THE DOCUMENT OR THE USE OR OTHER DEALINGS IN THE DOCUMENT.

Acknowledgments

OpenMM software and all related activities, such as this manual, are funded by the Simbios National Center for Biomedical Computing through the National Institutes of Health Roadmap for Medical Research, Grant U54 GM072970. Information on the National Centers can be found at http://nihroadmap.nih.gov/bioinformatics.

CHAPTER ONE

INTRODUCTION

This guide describes the internal architecture of the OpenMM library. It is targeted at developers who want to add features to OpenMM, either by modifying the core library directly or by writing plugins. If you just want to write applications that use OpenMM, you do not need to read this guide; the Users Manual tells you everything you need to know. This guide is *only* for people who want to contribute to OpenMM itself.

It is organized as follows:

- Chapter *2* describes the architecture of the core OpenMM library. It discusses how the high level and low level APIs relate to each other, and the flow of execution between them.
- Chapter *3* describes in detail how to write a plugin. It focuses on the two most common types of plugins: those which define new Forces, and those which implement new Platforms.
- Chapter *4* discusses the architecture of the reference Platform, providing information relevant to writing reference implementations of new features.
- Chapter *5* discusses the architecture of the CPU Platform, providing information relevant to writing CPU implementations of new features.
- Chapter *6* discusses the architecture of the OpenCL Platform, providing information relevant to writing OpenCL implementations of new features.
- Chapter *7* discusses the architecture of the CUDA Platform, providing information relevant to writing CUDA implementations of new features.

This guide assumes you are already familiar with the public API and how to use OpenMM in applications. If that is not the case, you should first read the Users Manual and work through some of the example programs. Pay especially close attention to the "Introduction to the OpenMM Library" chapter, since it introduces concepts that are important in understanding this guide.

CHAPTER

TWO

THE CORE LIBRARY

OpenMM is based on a layered architecture, as shown in the following diagram:

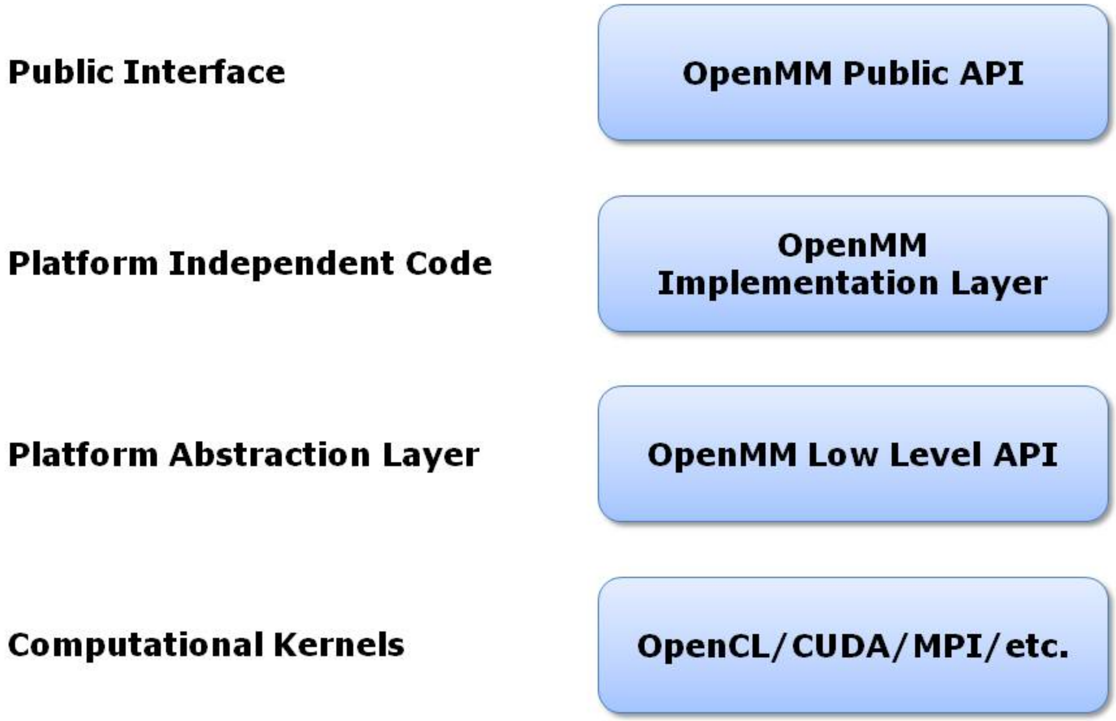

Figure 2-1: OpenMM architecture

The public API layer consists of the classes you access when using OpenMM in an application: System; Force and its subclasses; Integrator and its subclasses; and Context. These classes define a public interface but do no computation.

The next layer down consists of “implementation” classes that mirror the public API classes: ContextImpl, ForceImpl, and a subclass of ForceImpl for each subclass of Force (HarmonicBondForceImpl, NonbondedForceImpl, etc.). These objects are created automatically when you create a Context. They store information related to a particular simulation, and define methods for performing calculations.

Note that, whereas a Force is logically "part of" a System, a ForceImpl is logically "part of" a Context. (See Figure 2-2.) If you create many Contexts for simulating the same System, there is still only one System and only one copy of each Force in it. But there will be separate ForceImpls for each Context, and those ForceImpls store information related to their particular Contexts.

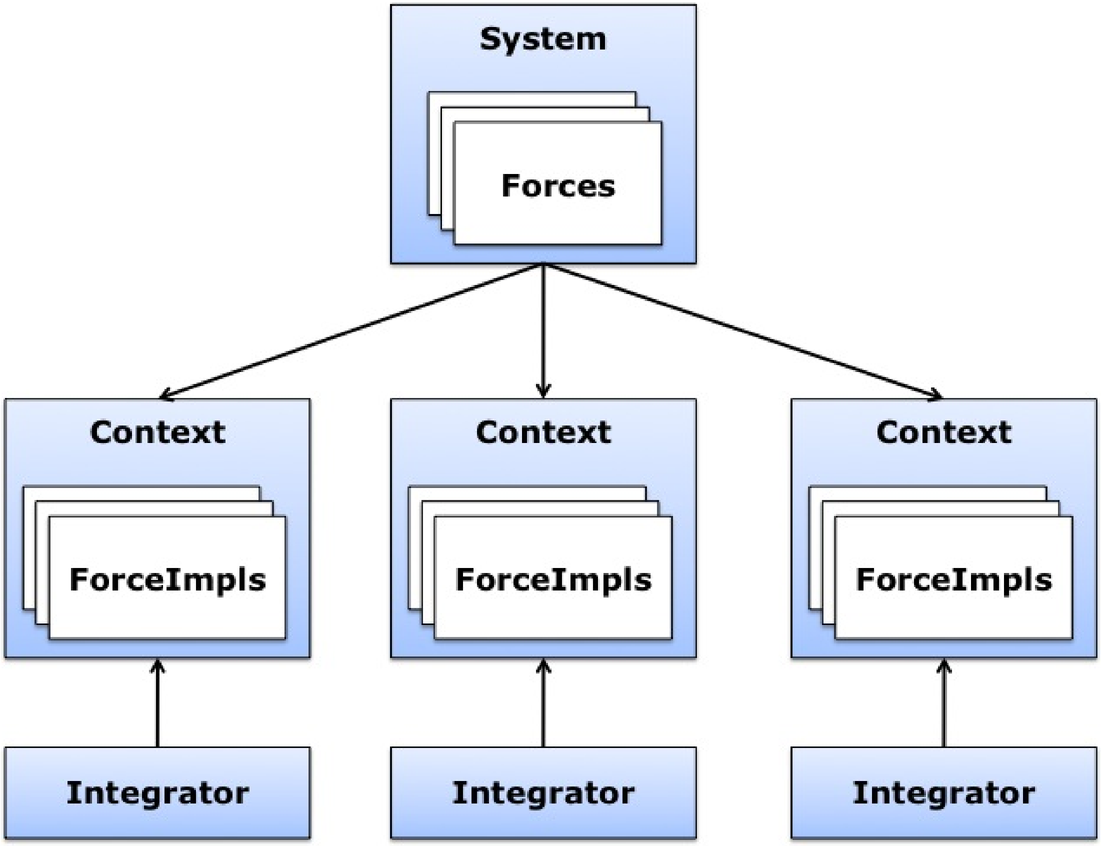

Figure 2-2: Relationships between public API and implementation layer objects

Also note that there is no "IntegratorImpl" class, because it is not needed. Integrator is already specific to one Context. Many Contexts can all simulate the same System, but each of them must have its own Integrator, so information specific to one simulation can be stored directly in the Integrator.

The next layer down is the OpenMM Low Level API (OLLA). The important classes in this layer are: Platform; Kernel; KernelImpl and its subclasses; and KernelFactory. A Kernel is just a reference counted pointer to a KernelImpl; the real work is done by KernelImpl objects (or more precisely, by instances of its subclasses). A KernelFactory creates KernelImpl objects, and a Platform ties together a set of KernelFactories, as well as defining information that applies generally to performing computations with that Platform.

All of these classes (except Kernel) are abstract. A particular Platform provides concrete subclasses of all of them. For example, the reference platform defines a Platform subclass called ReferencePlatform, a KernelFactory subclass called ReferenceKernelFactory, and a concrete subclass of each abstract KernelImpl type: ReferenceCalcNonbondedForceKernel extends CalcNonbondedForceKernel (which in turn extends KernelImpl), ReferenceIntegrateVerletStepKernel extends IntegrateVerletStepKernel, and so on.

We can understand this better by walking through the entire sequence of events that takes place when you create a Context. As an example, suppose you create a System; add a NonbondedForce to it; create a VerletIntegrator; and then

create a Context for them using the reference Platform. Here is what happens.

1. The Context constructor creates a ContextImpl.
2. The ContextImpl calls `createImpl()` on each Force in the System, which creates an instance of the appropriate ForceImpl subclass.
3. The ContextImpl calls `contextCreated()` on the Platform(), which in turn calls `setPlatformData()` on the ContextImpl. This allows Platform-specific information to be stored in a ContextImpl. Every Platform has its own mechanism for storing particle masses, constraint definitions, particle positions, and so on. ContextImpl therefore allows the Platform to create an arbitrary block of data and store it where it can be accessed by that Platform's kernels.
4. The ContextImpl calls `createKernel()` on the Platform several times to get instances of various kernels that it needs: CalcKineticEnergyKernel, ApplyConstraintsKernel, etc.
   (a) For each kernel, the Platform looks up which KernelFactory has been registered for that particular kernel. In this case, it will be a ReferenceKernelFactory.
   (b) It calls `createKernelImpl()` on the KernelFactory, which creates and returns an instance of an appropriate KernelImpl subclass: ReferenceCalcKineticEnergyKernel, ReferenceApplyConstraintsKernel, etc.
5. The ContextImpl loops over all of its ForceImpls and calls `initialize()` on each one.
   (a) Each ForceImpl asks the Platform to create whatever kernels it needs. In this example, NonbondedForceImpl will request a CalcNonbondedForceKernel, and get back a ReferenceCalcNonbondedForceKernel.
6. The ContextImpl calls `initialize()` on the Integrator which, like the other objects, requests kernels from the Platform. In this example, VerletIntegrator requests an IntegrateVerletStepKernel and gets back a ReferenceIntegrateVerletStepKernel.

At this point, the Context is fully initialized and ready for doing computation. Reference implementations of various KernelImpls have been created, but they are always referenced through abstract superclasses. Similarly, data structures specific to the reference Platform have been created and stored in the ContextImpl, but the format and content of these structures is opaque to the ContextImpl. Whenever it needs to access them (for example, to get or set particle positions), it does so through a kernel (UpdateStateDataKernel in this case).

Now suppose that you call `step()` on the VerletIntegrator. Here is what happens to execute each time step.

1. The VerletIntegrator calls `updateContextState()` on the ContextImpl. This gives each Force an opportunity to modify the state of the Context at the start of each time step.
   (a) The ContextImpl loops over its ForceImpls and calls `updateContextState()` on each one. In this case, our only ForceImpl is a NonbondedForceImpl, which returns without doing anything. On the other hand, if we had an AndersenThermostat in our System, its ForceImpl would invoke a kernel to modify particle velocities.
2. The VerletIntegrator calls `calcForcesAndEnergy()` on the ContextImpl to request that the forces be computed.
   (a) The ContextImpl calls `beginComputation()` on its CalcForcesAndEnergyKernel. This initializes all the forces to zero and does any other initialization the Platform requires before forces can be computed. For example, some Platforms construct their nonbonded neighbor lists at this point.
   (b) The ContextImpl loops over its ForceImpls and calls `calcForcesAndEnergy()` on each one. In this case, we have a NonbondedForceImpl which invokes its CalcNonbondedForceKernel to compute forces.
   (c) Finally, the ContextImpl calls `finishComputation()` on its CalcForcesAndEnergyKernel. This does any additional work needed to determine the final forces, such as summing the values from intermediate buffers.

3. Finally, the VerletIntegrator invokes its IntegrateVerletStepKernel. This takes the forces, positions, and velocities that are stored in a Platform- specific format in the ContextImpl, uses them to compute new positions and velocities, and stores them in the ContextImpl.

CHAPTER THREE

WRITING PLUGINS

A plugin is a dynamic library that adds new features to OpenMM. It is typically stored in the `lib/plugins` directory inside your OpenMM installation, and gets loaded along with all other plugins when the user calls

```
Platform::loadPluginsFromDirectory(Platform::getDefaultPluginsDirectory());
```

It is also possible to load plugins from a different directory, or to load them individually by calling `Platform::loadPluginLibrary()`.

Every plugin must implement two functions that are declared in the PluginInitializer.h header file:

```
extern "C" void registerPlatforms();
extern "C" void registerKernelFactories();
```

When a plugin is loaded, these two functions are invoked to register any Platforms and KernelFactories defined by the plugin. When many plugins are loaded at once by calling `Platform::loadPluginsFromDirectory()`, `registerPlatforms()` is first called on all of them, then `registerKernelFactories()` is called on all of them. This allows one plugin to define a Platform, and a different plugin to add KernelFactories to it; the Platform is guaranteed to be registered by the first plugin before the second plugin tries to add its KernelFactories, regardless of what order the plugins happen to be loaded in.

3.1 Creating New Platforms

One common type of plugin defines a new Platform. There are three such plugins that come with OpenMM: one for the CPU Platform, one for the CUDA Platform, and one for the OpenCL Platform.

To define a new Platform, you must create subclasses of the various abstract classes in the OpenMM Low Level API: a subclass of Platform, one or more subclasses of KernelFactory, and a subclass of each KernelImpl. That is easy to say, but a huge amount of work to actually do. There are many different algorithms involved in computing forces, enforcing constraints, performing integration, and so on, all of which together make up a Platform. Of course, there is no requirement that every Platform must implement every possible feature. If you do not provide an implementation of a particular kernel, it simply means your Platform cannot be used for any simulation that requires that kernel; if a user tries to do so, an exception will be thrown.

Your plugin's `registerPlatforms()` function should create an instance of your Platform subclass, then register it by calling `Platform::registerPlatform()`. You also must register the KernelFactory for each kernel your Platform supports. This can be done in the `registerKernelFactories()` function, or more simply, directly in the Platform's constructor. You can use as many different KernelFactories as you want for different kernels, but usually it is simplest to use a single KernelFactory for all of them. The support for multiple KernelFactories exists primarily to let plugins add new features to existing Platforms, as described in the next section.

3.2 Creating New Forces

Another common type of plugin defines new Forces and provides implementations of them for existing Platforms. (Defining new Integrators is not specifically discussed here, but the process is very similar.) There are two such plugins that come with OpenMM. They implement the AMOEBA force field and Drude oscillators, respectively.

As an example, suppose you want to create a new Force subclass called StringForce that uses the equations of String Theory to compute the interactions between particles. You want to provide implementations of it for all four standard platforms: Reference, CPU, CUDA, and OpenCL.

The first thing to realize is that this *cannot* be done with only a plugin library. Plugins are loaded dynamically at runtime, and they relate to the low level API; but you must also provide a public API. Users of your class need to create StringForce objects and call methods on them. That means providing a header file with the class declaration, and a (non-plugin) library with the class definition to link their code against. The implementations for particular Platforms can be in plugins, but the public API class itself cannot. Or to put it differently, the full "plugin" (from the user's perspective) consists of three parts: the library OpenMM loads at runtime (which is what OpenMM considers to be the "plugin"), a second library for users to link their code against, and a header file for them to include in their source code.

To define the API, you will need to create the following classes:

1. StringForce. This is the public API for your force, and users will directly link against the library containing it.
2. StringForceImpl. This is the ForceImpl subclass corresponding to StringForce. It should be defined in the same library as StringForce, and StringForce's `createImpl()` method should create an instance of it.
3. CalcStringForceKernel. This is an abstract class that extends KernelImpl, and defines the API by which StringForceImpl invokes its kernel. You only need to provide a header file for it, not an implementation; those will be provided by Platforms.

Now suppose you are writing the OpenCL implementation of StringForce. Here are the classes you need to write:

1. OpenCLCalcStringForceKernel. This extends CalcStringForceKernel and provides implementations of its virtual methods. The code for this class will probably be very complicated (and if it actually works, worth a Nobel Prize). It may execute many different GPU kernels and create its own internal data structures. But those details are entirely internal to your own code. As long as this class implements the virtual methods of CalcStringForceKernel, you can do anything you want inside it.
2. OpenCLStringForceKernelFactory. This is a KernelFactory subclass that knows how to create instances of OpenCLCalcStringForceKernel.

Both of these classes should be packaged into a dynamic library (.so on Linux, .dylib on Mac, .dll on Windows) that can be loaded as a plugin. This library must also implement the two functions from PluginInitializer.h. `registerPlatforms()` will do nothing, since this plugin does not implement any new Platforms. `registerKernelFactories()` should call `Platform::getPlatformByName("OpenCL")` to get the OpenCL Platform, then create a new OpenCLStringForceKernelFactory and call `registerKernelFactory()` on the Platform to register it. If the OpenCL Platform is not available, you should catch the exception then return without doing anything. Most likely this means there is no OpenCL runtime on the computer your code is running on.

CHAPTER FOUR

THE REFERENCE PLATFORM

The reference Platform is written with simplicity and clarity in mind, not performance. (It is still not always as simple or clear as one might hope, but that is the goal.) When implementing a new feature, it is recommended to create the reference implementation first, then use that as a model for the versions in other Platforms.

The reference Platform represents all floating point numbers with the type RealOpenMM, which is defined in SimTKOpenMMRealType.h. This allows the entire platform to be compiled in either single or double precision. By default it is double precision, but it can be changed by modifying one flag at the top of that file. The same file also defines lots of numerical constants and mathematical functions, so the correct precision version will always be used. Vector quantities (positions, velocities, etc.) are represented by RealVec objects. This class is identical to Vec3, except that its components are of type RealOpenMM instead of double.

When using the reference Platform, the “platform-specific data” stored in ContextImpl is of type ReferencePlatform::PlatformData, which is declared in ReferencePlatform.h. Several of the fields in this class are declared as void* to avoid having to include SimTKOpenMMRealType.h in ReferencePlatform.h. If you look in ReferenceKernels.cpp, you will find code for extracting the correct values of these fields. For example:

```
static vector<RealVec>& extractPositions(ContextImpl& context) {
    ReferencePlatform::PlatformData* data =
            reinterpret_cast<ReferencePlatform::PlatformData*>(context.getPlatformData());
    return *((vector<RealVec>*) data->positions);
}
```

The PlatformData’s vector of forces contains one element for each particle. At the start of each force evaluation, all elements of it are set to zero. Each Force adds its own contributions to the vector, so that at the end, it contains the total force acting on each particle.

There are a few additional classes that contain useful static methods. SimTKOpenMMUtilities has various utility functions, of which the most important is a random number generator. ReferenceForce provides methods for calculating the displacement between two positions, optionally taking periodic boundary conditions into account.

CHAPTER

FIVE

THE CPU PLAFORM

CpuPlatform is a subclass of ReferencePlatform. It provides optimized versions of a small number of kernels, while using the reference implementations for all the others. Any kernel implementation written for the reference Platform will work equally well with the CPU platform. Of course, if that kernel happens to be a performance bottleneck, you will probably want to write an optimized version of it. But many kernels have negligible effect on performance, and for these you can just use the same implementation for both platforms.

If you choose to do that, you can easily support both platforms with a single plugin library. Just implement `registerKernelFactories()` like this:

```
extern "C" void registerKernelFactories() {
    for (int i = 0; i < Platform::getNumPlatforms(); i++) {
        Platform& platform = Platform::getPlatform(i);
        if (dynamic_cast<ReferencePlatform*>(&platform) != NULL) {
            // Create and register your KernelFactory.
        }
    }
}
```

The loop identifies every ReferencePlatform, either an instance of the base class or of a subclass, and registers a KernelFactory for every one.

CHAPTER

SIX

THE OPENCL PLATFORM

The OpenCL Platform is much more complicated than the reference Platform. It also provides many more tools to simplify your work, but those tools themselves can be complicated to use correctly. This chapter will attempt to explain how to use some of the most important ones. It will *not* teach you how to program with OpenCL. There are many tutorials on that subject available elsewhere, and this guide assumes you already understand it.

6.1 Overview

When using the OpenCL Platform, the "platform-specific data" stored in ContextImpl is of type OpenCLPlatform::PlatformData, which is declared in OpenCLPlatform.h. The most important field of this class is `contexts` , which is a vector of OpenCLContexts. (There is one OpenCLContext for each device you are using. The most common case is that you are running everything on a single device, in which case there will be only one OpenCLContext. Parallelizing computations across multiple devices is not discussed here.) The OpenCLContext stores most of the important information about a simulation: positions, velocities, forces, an OpenCL CommandQueue used for executing kernels, workspace buffers of various sorts, etc. It provides many useful methods for compiling and executing kernels, clearing and reducing buffers, and so on. It also provides access to three other important objects: the OpenCLIntegrationUtilities, OpenCLNonbondedUtilities, and OpenCLBondedUtilities. These are discussed below.

Allocation of device memory is generally done through the OpenCLArray class. It takes care of much of the work of memory management, and provides a simple interface for transferring data between host and device memory.

Every kernel is specific to a particular OpenCLContext, which in turn is specific to a particular OpenMM::Context. This means that kernel source code can be customized for a particular simulation. For example, values such as the number of particles can be turned into compile-time constants, and specific versions of kernels can be selected based on the device being used or on particular aspects of the system being simulated. `OpenCLContext::createProgram()` makes it easy to specify a list of preprocessor definitions to use when compiling a kernel.

The normal way to execute a kernel is by calling `executeKernel()` on the OpenCLContext. It allows you to specify the total number of work-items to execute, and optionally the size of each work-group. (If you do not specify a work-group size, it uses 64 as a default.) The number of work-groups to launch is selected automatically based on the work-group size, the total number of work-items, and the number of compute units in the device it will execute on.

6.2 Numerical Precision

The OpenCL platform supports three precision modes:

1. **Single**: All values are stored in single precision, and nearly all calculations are done in single precision. The arrays of positions, velocities, forces, and energies (returned by the OpenCLContext's `getPosq()`, `getVelm()`, `getForce()`, `getForceBuffers()`, and `getEnergyBuffer()` methods) are all of type `float4` (or `float` in the case of `getEnergyBuffer()`).

2. **Mixed**: Forces are computed and stored in single precision, but integration is done in double precision. The velocities have type `double4`. The positions are still stored in single precision to avoid adding overhead to the force calculations, but a second array of type `float4` is created to store "corrections" to the positions (returned by the OpenCLContext's getPosqCorrection() method). Adding the position and the correction together gives the full double precision position.

3. **Double**: Positions, velocities, forces, and energies are all stored in double precision, and nearly all calculations are done in double precision.

You can call `getUseMixedPrecision()` and `getUseDoublePrecision()` on the OpenCLContext to determine which mode is being used. In addition, when you compile a kernel by calling `createKernel()`, it automatically defines two types for you to make it easier to write kernels that work in any mode:

1. `real` is defined as `float` in single or mixed precision mode, `double` in double precision mode.

2. `mixed` is defined as `float` in single precision mode, `double` in mixed or double precision mode.

It also defines vector versions of these types (`real2`, `real4`, etc.).

6.3 Computing Forces

When forces are computed, they are stored in multiple buffers. This is done to enable multiple work-items or work-groups to compute forces on the same particle at the same time; as long as each one writes to a different buffer, there is no danger of race conditions. At the start of a force calculation, all forces in all buffers are set to zero. Each Force is then free to add its contributions to any or all of the buffers. Finally, the buffers are summed to produce the total force on each particle.

The size of each buffer is equal to the number of particles, rounded up to the next multiple of 32. Call `getPaddedNumAtoms()` on the OpenCLContext to get that number. The actual force buffers are obtained by calling `getForceBuffers()`. The first *n* entries (where *n* is the padded number of atoms) represent the first force buffer, the next *n* represent the second force buffer, and so on. More generally, the *i*'th force buffer's contribution to the force on particle *j* is stored in element `i*context.getPaddedNumAtoms()+j`.

Depending on the device, a buffer may also be created that stores contributions to the forces in 64 bit fixed point format. On devices that support atomic operations on 64 bit integers in global memory, this can be a more efficient way of accumulating forces than using a large number of force buffers. To convert a value from floating point to fixed point, multiply it by 0x100000000 (2^{32}), then cast it to a `long`. The fixed point buffer is ordered differently from the others. For atom *i*, the x component of its force is stored in element `i`, the y component in element `i+context.getPaddedNumAtoms()`, and the z component in element `i+2*context.getPaddedNumAtoms()`.

The potential energy is also accumulated in a set of buffers, but this one is simply a list of floating point values. All of them are set to zero at the start of a computation, and they are summed at the end of the computation to yield the total energy.

The OpenCL implementation of each Force object should define a subclass of OpenCLForce, and register an instance of it by calling `addForce()` on the OpenCLContext. This serves two purposes:

1. It reports how many force buffers are required when calculating this particular Force. The OpenCLContext sets the size of its force buffer array based on the largest number of buffers required by any Force.

2. It implements methods for determining whether particular particles or groups of particles are identical. This is important when reordering particles, and is discussed below.

6.4 Nonbonded Forces

Computing nonbonded interactions efficiently is a complicated business in the best of cases. It is even more complicated on a GPU. Furthermore, the algorithms must vary based on the type of processor being used, whether there is a distance cutoff, and whether periodic boundary conditions are being applied.

The OpenCLNonbondedUtilities class tries to simplify all of this. To use it you need provide only a piece of code to compute the interaction between two particles. It then takes responsibility for generating a neighbor list, looping over interacting particles, loading particle parameters from global memory, and writing the forces and energies to the appropriate buffers. All of these things are done using an algorithm appropriate to the processor you are running on and high level aspects of the interaction, such as whether it uses a cutoff and whether particular particle pairs need to be excluded.

Of course, this system relies on certain assumptions, the most important of which is that the Force can be represented as a sum of independent pairwise interactions. If that is not the case, things become much more complicated. You may still be able to use features of OpenCLNonbondedUtilities, but you cannot use the simple mechanism outlined above. That is beyond the scope of this guide.

To define a nonbonded interaction, call `addInteraction()` on the OpenCLNonbondedUtilities, providing a block of OpenCL source code for computing the interaction. This block of source code will be inserted into the middle of an appropriate kernel. At the point where it is inserted, various variables will have been defined describing the interaction to compute:

1. `atom1` and `atom2` are the indices of the two interacting particles.
2. `r`, `r2`, and `invR` are the distance r between the two particles, r^2, and $1/r$ respectively.
3. `isExcluded` is a `bool` specifying whether this pair of particles is marked as an excluded interaction. (Excluded pairs are not skipped automatically, because in some cases they still need to be processed, just differently from other pairs.)
4. `posq1` and `posq2` are `real4`s containing the positions (in the xyz fields) and charges (in the w fields) of the two particles.
5. Other per-particle parameters may be specified, as described below.

The following preprocessor macros will also have been defined:

1. `NUM_ATOMS` is the total number of particles in the system.
2. `PADDED_NUM_ATOMS` is the padded number of particles in the system.
3. `USE_CUTOFF` is defined if and only if a cutoff is being used
4. `USE_PERIODIC` is defined if and only if periodic boundary conditions are being used.
5. `CUTOFF` and `CUTOFF_SQUARED` are the cutoff distance and its square respectively (but only defined if a cutoff is being used).

Finally, two output variables will have been defined:

1. You should add the energy of the interaction to `tempEnergy`.
2. You should add the derivative of the energy with respect to the inter-particle distance to `dEdR`.

You can also define arbitrary per-particle parameters by calling `addParameter()` on the OpenCLNonbondedUtilities. You provide an array in device memory containing the set of values, and the values for the two interacting particles will be loaded and stored into variables called `<name>1` and `<name>2`, where <name> is the name you specify for the parameter. Note that nonbonded interactions are not computed until after `calcForcesAndEnergy()` has been called on every ForceImpl, so it is possible to make the parameter values change with time by modifying them inside `calcForcesAndEnergy()`. Also note that the length of the array containing the parameter values must equal the *padded* number of particles in the system.

Finally, you can specify arbitrary other memory objects that should be passed as arguments to the interaction kernel by calling `addArgument()`. The rest of the kernel ignores these arguments, but you can make use of them in your interaction code.

Consider a simple example. Suppose we want to implement a nonbonded interaction of the form $E=k_1k_2r^2$, where k is a per-particle parameter. First we create a parameter as follows

```
nb.addParameter(OpenCLNonbondedUtilities::ParameterInfo("kparam", "float", 1,
        sizeof(cl_float), kparam->getDeviceBuffer()));
```

where `nb` is the OpenCLNonbondedUtilities for the context. Now we call `addInteraction()` to define an interaction with the following source code:

```
#ifdef USE_CUTOFF
if (!isExcluded && r2 < CUTOFF_SQUARED) {
#else
if (!isExcluded) {
#endif
    tempEnergy += kparam1*kparam2*r2;
    dEdR += 2*kparam1*kparam2*r;
}
```

An important point is that this code is executed for every pair of particles in the *padded* list of atoms. This means that some interactions involve padding atoms, and should not actually be included. You might think, then, that the above code is incorrect and we need another check to filter out the extra interactions:

```
if (atom1 < NUM_ATOMS && atom2 < NUM_ATOMS)
```

This is not necessary in our case, because the `isExcluded` flag is always set for interactions that involve a padding atom. If our force did not use excluded interactions (and so did not check `isExcluded`), then we would need to add this extra check. Self interactions are a similar case: we do not check for `(atom1 == atom2)` because the exclusion flag prevents them from being processed, but for some forces that check is necessary.

6.5 Bonded Forces

Just as OpenCLNonbondedUtilities simplifies the task of creating nonbonded interactions, OpenCLBondedUtilities simplifies the process for many types of bonded interactions. A "bonded interaction" means one that is applied to small, fixed groups of particles. This includes bonds, angles, torsions, etc. The important point is that the list of particles forming a "bond" is known in advance and does not change with time.

Using OpenCLBondedUtilities is very similar to the process described above. You provide a block of OpenCL code for evaluating a single interaction. This block of code will be inserted into the middle of a kernel that loops over all interactions and evaluates each one. At the point where it is inserted, the following variables will have been defined describing the interaction to compute:

1. `index` is the index of the interaction being evaluated.
2. `atom1`, `atom2`, ... are the indices of the interacting particles.
3. `pos1`, `pos2`, ... are `real4`s containing the positions (in the xyz fields) of the interacting particles.

A variable called `energy` will have been defined for accumulating the total energy of all interactions. Your code should add the energy of the interaction to it. You also should define `real4` variables called `force1`, `force2`, ... and store the force on each atom into them.

As a simple example, the following source code implements a pairwise interaction of the form $E=r^2$:

```
real4 delta = pos2-pos1;
energy += delta.x*delta.x + delta.y*delta.y + delta.z*delta.z;
real4 force1 = 2.0f*delta;
real4 force2 = -2.0f*delta;
```

To use it, call `addInteraction()` on the Context's OpenCLBondedUtilities object. You also provide a list of the particles involved in every bonded interaction.

Exactly as with nonbonded interactions, you can call `addArgument()` to specify arbitrary memory objects that should be passed as arguments to the interaction kernel. These might contain per-bond parameters (use `index` to look up the appropriate element) or any other information you want.

6.6 Reordering of Particles

Nonbonded calculations are done a bit differently in the OpenCL Platform than in most CPU based codes. In particular, interactions are computed on blocks of 32 particles at a time (which is why the number of particles needs to be padded to bring it up to a multiple of 32), and the neighbor list actually lists pairs of *blocks*, not pairs of individual particles, that are close enough to interact with each other.

This only works well if sequential particles tend to be close together so that blocks are spatially compact. This is generally true of particles in a macromolecule, but it is not true for solvent molecules. Each water molecule, for example, can move independently of other water molecules, so particles that happen to be sequential in whatever order the molecules were defined in need not be spatially close together.

The OpenCL Platform addresses this by periodically reordering particles so that sequential particles are close together. This means that what the OpenCL Platform calls particle *i* need not be the same as what the System calls particle *i*.

This reordering is done frequently, so it must be very fast. If all the data structures describing the structure of the System and the Forces acting on it needed to be updated, that would make it prohibitively slow. The OpenCL Platform therefore only reorders particles in ways that do not alter any part of the System definition. In practice, this means exchanging entire molecules; as long as two molecules are truly identical, their positions and velocities can be exchanged without affecting the System in any way.

Every Force can contribute to defining the boundaries of molecules, and to determining whether two molecules are identical. This is done through the OpenCLForceInfo it adds to the OpenCLContext. It can specify two types of information:

1. Given a pair of particles, it can say whether those two particles are identical (as far as that Force is concerned). For example, a Force object implementing a Coulomb force would check whether the two particles had equal charges.
2. It can define *particle groups*. The OpenCL Platform will ensure that all the particles in a group are part of the same molecule. It also can specify whether two groups are identical to each other. For example, in a Force implementing harmonic bonds, each group would consist of the two particles connected by a bond, and two groups would be identical if they had the same spring constants and equilibrium lengths.

6.7 Integration Utilities

The OpenCLContext's OpenCLIntegrationUtilities provides features that are used by many integrators. The two most important are random number generation and constraint enforcement.

If you plan to use random numbers, you should call `initRandomNumberGenerator()` during initialization, specifying the random number seed to use. Be aware that there is only one random number generator, even if multiple classes make use of it. If two classes each call `initRandomNumberGenerator()` and request different seeds, an exception will be thrown. If they each request the same seed, the second call will simply be ignored.

For efficiency, random numbers are generated in bulk and stored in an array in device memory, which you can access by calling `getRandom()`. Each time you need to use a block of random numbers, call `prepareRandomNumbers()`, specifying how many values you need. It will register that many values as having been used, and return the index in the array at which you should start reading values. If not enough unused values remain in the array, it will generate a new batch of random values before returning.

To apply constraints, simply call `applyConstraints()`. For numerical accuracy, the constraint algorithms do not work on particle positions directly, but rather on the *displacements* taken by the most recent integration step. These displacements must be stored in an array which you can get by calling `getPosDelta()`. That is, the constraint algorithms assume the actual (unconstrained) position of each particle equals the position stored in the OpenCLContext plus the delta stored in the OpenCLIntegrationUtilities. It then modifies the deltas so that all distance constraints are satisfied. The integrator must then finish the time step by adding the deltas to the positions and storing them into the main position array.

CHAPTER

SEVEN

THE CUDA PLATFORM

The CUDA platform is very similar to the OpenCL platform, and most of the previous chapter applies equally well to it, just changing "OpenCL" to "Cuda" in class names. There are a few differences worth noting.

7.1 Compiling Kernels

Like the OpenCL platform, the CUDA platform compiles all its kernels at runtime. Unlike OpenCL, CUDA does not have built in support for runtime compilation. OpenMM therefore needs to implement this itself by writing the source code out to disk, invoking the nvcc compiler as a separate process, and then loading the compiled kernel in from disk.

For the most part, you can ignore all of this. Just call `createModule()` on the CudaContext, passing it the CUDA source code. It takes care of the details of compilation and loading, returning a CUmodule object when it is done. You can then call `getKernel()` to look up individual kernels in the module (represented as CUfunction objects) and `executeKernel()` to execute them.

The CUDA platform does need two things to make this work: a directory on disk where it can write out temporary files, and the path to the nvcc compiler. These are specified by the "CudaTempDirectory" and "CudaCompiler" properties when you create a new Context. It often can figure out suitable values for them on its own, but sometimes it needs help. See the "Platform-Specific Properties" chapter of the Users Manual for details.

7.2 Accumulating Forces

The OpenCL platform, as described in Section *6.3*, uses two types of buffers for accumulating forces: a set of floating point buffers, and a single fixed point buffer. In contrast, the CUDA platform uses *only* the fixed point buffer (represented by the CUDA type `long long`). This means the CUDA platform only works on devices that support 64 bit atomic operations (compute capability 1.2 or higher).
